# Cardiac amyloid presenting as ST-elevation myocardial infarct: A case of amyloid induced coronary vasospasm

**DOI:** 10.1016/j.hrcr.2025.06.019

**Published:** 2025-08-15

**Authors:** Sarah R. Gutierrez, Monika M. Do, David F. Meoli

**Affiliations:** 1 The Department of Veterans Affairs, Tennessee Valley Health System, Nashville, Tennessee; 2 The Department of Veterans Affairs, Tennessee Valley Health System and Vanderbilt University Medical Center, Nashville, Tennessee

**Keywords:** Cardiac amyloid, Vasospasm, ST-elevation myocardial infarction, Chest pain, TTR amyloidosis

## Introduction

Cardiac amyloidosis refers to the pathologic deposition of amyloid fibrils, composed of misfolded proteins, within the extracellular matrix of the heart.^1^ Cardiac amyloidosis is a known etiology of restrictive cardiomyopathy and may present with the characteristic electrocardiogram (ECG) findings such as low-voltage QRS complexes and pseudoinfarct patterns in the absence of coronary artery disease.^2^ These findings result from impaired myocardial conduction secondary to amyloid infiltration. Impaired microvascular perfusion can produce dynamic ST-segment and T-wave abnormalities consistent with myocardial ischemia.^3^ Coronary vasospasm is characterized by transient, reversible constriction of the coronary arteries, leading to reduced myocardial blood flow and subsequent ischemia.^3^ This phenomenon can precipitate angina in the absence of atherosclerotic plaque. This case report highlights ECG changes associated with elevated troponin I levels, triggered by coronary vasospasm secondary to cardiac amyloidosis. Understanding the pathophysiology, clinical presentation, and management of this phenomenon is essential for cardiology providers.

## Case report

A 63-year-old African American male presented to a quaternary care center with precordial chest pain under an ST-elevation myocardial infarction alert protocol. His medical history included coronary artery disease with previous right coronary artery stenting and coronary artery bypass grafting involving the left internal mammary artery to the left anterior descending artery, saphenous vein grafts to the obtuse marginal, and the posterior descending artery. He had known heart failure with reduced ejection fraction, mitral regurgitation, hypertension, hyperlipidemia, type 2 diabetes mellitus, and newly diagnosed transthyretin (TTR) amyloidosis 3 months earlier confirmed by technetium-99m pyrophosphate scan showing myocardial uptake indicative of amyloid deposition.

**Figure 1:**
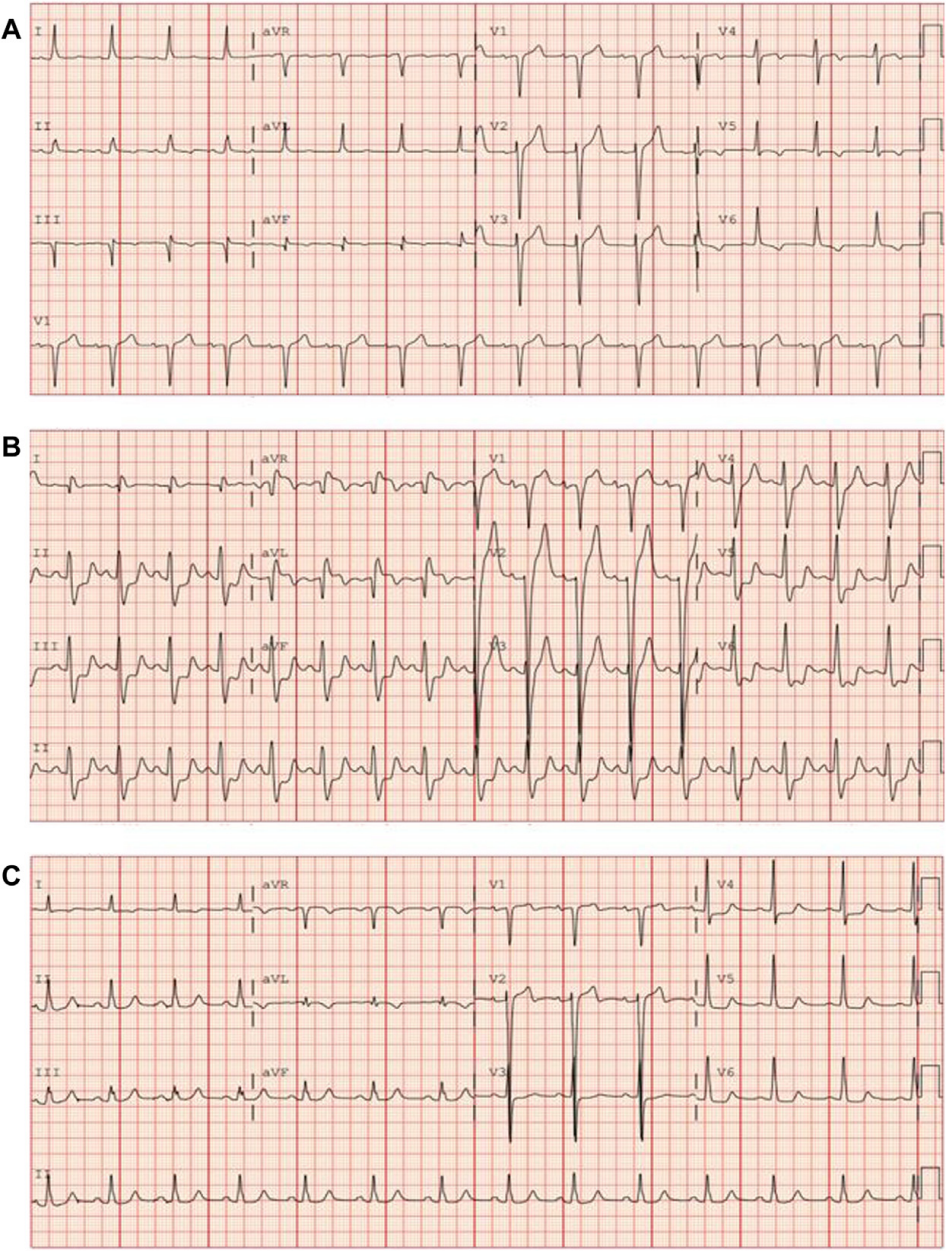
**A:** Baseline ECG. **B:** ECG on arrival showing acute ST elevation. **C:** ECG 24 hours after event. ECG = electrocardiogram.

Baseline ECG is presented in Figure 1A. On arrival, ECG (Figure 1B) revealed ST elevations in the anterior leads with reciprocal depressions in the lateral precordial and inferior leads. Troponin I peaked at 24.16 ng/mL. Emergent left coronary angiography showed patent grafts and severe native vessel disease, but no culprit lesion or thrombus. No intervention was performed. His chest pain resolved within 12 hours, and ECG findings improved by 24 hours (Figure 1C). A transthoracic echocardiogram obtained at presentation showed a left ventricular ejection fraction (LVEF) of 15%–20%. A repeat transthoracic echocardiogram performed 48 hours later showed improved LVEF of 30%–40%. The patient had not yet started tafamidis at the time of admission.

## Discussion

Amyloid infiltration in small coronary arterioles can cause microvascular obstruction, leading to ischemic ECG changes that mimic ST-elevation myocardial infarction despite the absence of atherosclerotic disease. In patients with restrictive physiology from amyloidosis, myocardial stiffness reduces the ability of the heart walls to adapt to changes in blood flow, increasing the risk of coronary vasospasm. Anginal symptoms owing to intramural coronary artery obstruction during a vasospastic episode can be as severe as those of the patient presented earlier. The patient’s acute resolution of chest pain and ST-segment changes within 48 hours, along with confirmed preserved graft patency, suggest vasospasm or myocardial stunning rather than infarction. In addition, the subsequent LVEF improvement suggests a nonischemic etiology contributing to the acute episode. Of note, the increase in LVEF over 48 hours is atypical for progressive amyloidosis, which generally causes a decline in systolic function over time. Although improved LVEF may reflect transient myocardial dysfunction rather than fixed ischemia, this must be interpreted with caution. The timing between echocardiograms is important, and treatment variables (eg, beta-blockers, vasodilators, fluid status) may influence findings. The patient had not yet received tafamidis, a TTR stabilizer known to slow disease progression.^4^ Given the association between amyloidosis and endothelial dysfunction, coronary vasospasm should be considered as a potential mechanism of ischemia in patients with cardiac amyloidosis.

## Conclusion

This case highlights the diagnostic challenge of chest pain in patients with cardiac amyloidosis. Coronary vasospasms should be considered in patients with known amyloidosis presenting with ST elevations and nonobstructive coronary angiography. Management included supportive care and the initiation of vasodilators such as calcium channel blockers and nitrates, which led to the resolution of symptoms. Given the underlying diagnosis of TTR amyloidosis, the patient was evaluated for initiation of tafamidis, a Food and Drug Administration–approved disease-modifying treatment for TTR amyloidosis.^4^ A comprehensive diagnostic approach, including coronary imaging, ECGs, and functional assessments, is essential to differentiate between ischemic and nonischemic causes in this complex patient population.

## Disclosures

The authors have no conflicts of interest to disclose.
